# Cocoa-Rich Chocolate and Quality of Life in Postmenopausal Women: A Randomized Clinical Trial

**DOI:** 10.3390/nu12092754

**Published:** 2020-09-10

**Authors:** Irene A. Garcia-Yu, Luis Garcia-Ortiz, Manuel A. Gomez-Marcos, Emiliano Rodriguez-Sanchez, Olaya Tamayo-Morales, Jose A. Maderuelo-Fernandez, Jose I. Recio-Rodriguez

**Affiliations:** 1 Instituto de Investigación Biomédica de Salamanca (IBSAL), Unidad de Investigación de Atención Primaria de Salamanca (APISAL), Servicio de Salud de Castilla y León (SACyL), 37005 Salamanca, Spain; lgarciao@usal.es (L.G.-O.); magomez@usal.es (M.A.G.-M.); emiliano@usal.es (E.R.-S.); oul630@hotmail.com (O.T.-M.); jmaderuelo@saludcastillayleon.es (J.A.M.-F.); donrecio@usal.es (J.I.R.-R.); 2 Departamento de Ciencias Biomédicas y del Diagnóstico, Universidad de Salamanca, 37007 Salamanca, Spain; 3 Departamento de Medicina, Universidad de Salamanca, 37007 Salamanca, Spain; 4 Departamento de Enfermería y Fisioterapia, Universidad de Salamanca, 37007 Salamanca, Spain

**Keywords:** chocolate, postmenopause, quality of life, randomised controlled trial

## Abstract

Menopause has a negative impact on quality of life (QoL). The aim of the present study was to analyse the effect on QoL of adding 10 g per day of chocolate with a high concentration of cocoa (99%) to the habitual diet, for 6 months, in a sample of postmenopausal women. Postmenopausal women (*n* = 140) aged 50–64 years were randomised to either an addition of 10 g per day of cocoa-rich chocolate to their usual diet or no supplement addition. All variables were measured at baseline and after six months of intervention. QoL was evaluated using the 3-level version of EuroQol-5D (EuroQoL-5D-3L), the EuroQol Visual Analogue Scale (EQ-VAS) and the Cervantes scale. Analysis of covariance (ANCOVA) analyses adjusted for the main determinants of QoL considered in this study showed no changes in the global score of QoL evaluated with the EuroQoL-5D-3L. The intervention group showed an increase of 6.0 points (95% confidence interval (CI): 0.4, 11.7) in the EQ-VAS compared to the control group (*p* = 0.036). No significant changes were observed between groups in the global score of QoL nor in the dimensions and subdimensions measured with the Cervantes scale. The additional daily contribution of 10 g of cocoa-rich chocolate in postmenopausal women could have a slight impact on their perception toward their health state, although without modifying the health-related QoL or the dimensions that compose it.

## 1. Introduction

Menopause has a negative impact on quality of life (QoL), with a gradual decrease from the premenopausal period to the postmenopausal period [1], in terms of both physical and mental health [2,3]. Such decrease in the QoL of postmenopausal women is associated with the appearance of genitourinary [4,5,6] and, especially, vasomotor [1] symptoms, such as hot flushes [7]. Moreover, in many cases, other psychological factors appear together during the postmenopausal period, such as depression, which worsen the perception toward QoL [8].

The consumption of chocolate, especially dark chocolate, has been associated with small, yet beneficial changes in the cardiovascular health of postmenopausal women [9]. However, very few studies have analysed its relationship with mental health and/or components of QoL. Balboa-Castillo et al. [10] analysed a cohort of 4599 individuals (average age: 54.1 years, 50.8% women), with no evidence of correlation between QoL and a greater or lower consumption of 10 g/day of chocolate, although these authors did not include an analysis based on age and sex. In women, the consumption of chocolate could have a significant positive impact on sexual function, especially on sexual desire [11]. A greater consumption of chocolate has been associated with a higher score in the Centre for Epidemiologic Studies Depression Scale (CES-D) [12], in which 16 or more points often represent a positive screening result, although no cause–effect relationship has been established between showing more signs of depression and a greater consumption of chocolate [13].

The benefits of dark chocolate have been attributed to a type of polyphenols known as flavonoids, which include flavonols and other polyphenols, such as epicatechin and catechin. In this line, some studies [14] have related the consumption of certain flavonol- and polyphenol-rich products, such as propolis [15], fruits and vegetables [16], to QoL, with rather inconclusive results. In women, the habitual consumption of coffee has been associated with slight improvement in the mental dimension of QoL [17]. Similarly, in postmenopausal women, the consumption of fermented soy has been related to an improvement in QoL [18], whereas the Mediterranean diet, which includes a large variety of polyphenol-rich products, has not been clearly associated with an improvement in QoL in older adults [19].

To sum up, despite the worsening of QoL that takes place during menopause and the indications about the possible positive effect that the consumption of chocolate can have on such deterioration, there are very few studies that approached this topic, obtaining divergent and poorly clarifying results [10,11,13]. Beyond the evaluation of pharmacological and/or nutritional therapies during menopause, it is fundamental to determine the real impact of these on QoL [20].

This study aimed to assess the effect of adding 10 g per day of chocolate with a high concentration of cocoa (99%) to the habitual diet, for 6 months, on QoL, in a sample of postmenopausal women.

## 2. Materials and Methods

### 2.1. Design and Setting

This is a controlled, randomised clinical trial with two parallel groups. The participants were recruited in the doctor offices of four urban primary care centres of Salamanca (Spain) through consecutive sampling. The evaluation period was from June 2018 to August 2019. It was conducted in the Primary Care Research Unit of Salamanca (APISAL), which is part of the Spanish Research Network for Preventive Activities and Health Promotion in Primary Care (redIAPP) and the Institute of Biomedical Research of Salamanca (IBSAL). The clinical trial has been registered in clinicaltrials.gov as NCT03492983. The protocol of the trial has been published [21].

This manuscript presents results on quality of life as a secondary outcome from the clinical trial. Results on blood pressure, as the main outcome of the trial [22], as well as results on body composition, as a secondary outcome [23], have been previously published.

### 2.2. Study Participants

The study included 140 women aged 50–64 years in the postmenopausal period, defined as amenorrhea for at least 12 consecutive months. The exclusion criteria were: personal history of cardiovascular disease, diabetes mellitus, arterial hypertension or dyslipidaemia under pharmacological treatment, hypocaloric diets, clinically demonstrable neurological and/or neuropsychological disease, hormone replacement therapy, habitual consumption of over 210 g per week (g/week) of cocoa and intolerance and/or allergic reaction to cocoa or any of components of the supplement.

### 2.3. Sample Size

Sample size estimation was carried out considering the change in arterial systolic pressure as the main variable of this clinical trial. To detect a minimum difference of 2.9 mm Hg in systolic arterial pressure between the two groups, 140 participants were required (70 per group), assuming an alpha risk of 0.05, a beta risk of 0.20 in a two-sided contrast and a standard deviation (SD) of 5.8 mm Hg. A follow-up loss rate of 10% was also assumed. These calculations were based on the results obtained in a similar study that reported a decrease of 6.5 ± 5.8 mm Hg in systolic arterial pressure [24]. With the 140 participants included in this study, we obtained a power of 72% for the hypothesis test to detect a statistically significant difference of 6 points in the mean score of the EuroQol Visual Analogue Scale (EQ-VAS) between study groups, assuming an alpha risk of 0.05 in a two-sided contrast.

### 2.4. Procedures and Randomisation

**Figure 1:**
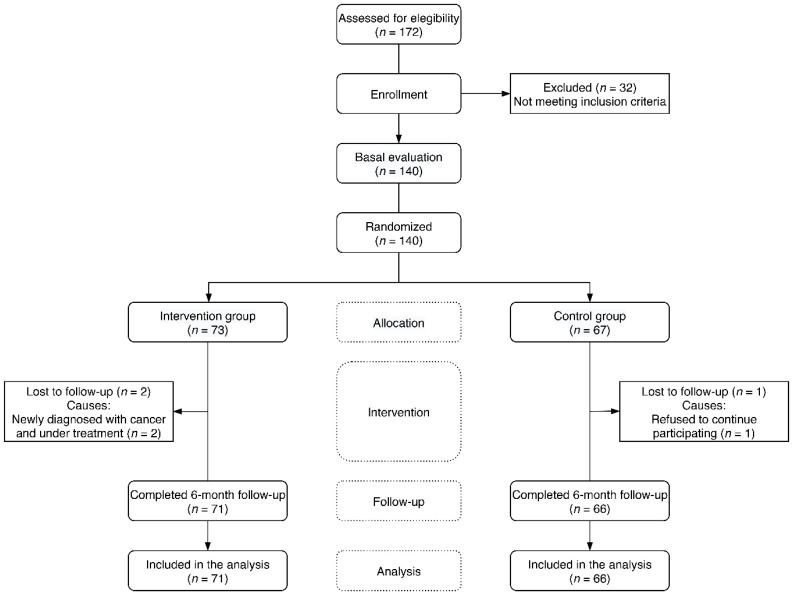
Flow diagram of postmenopausal women through the study.

The study variables were measured in all participants at baseline and at 6-month follow-up (Figure 1). The women in the intervention group paid another 5 visits for chocolate resupply at 1, 2, 3, 4 and 5 months after the baseline visit. In these resupply visits, held in the health centre, no other procedure was carried out, except for the supply of the necessary chocolate until the next visit and the gathering of a calendar with the record of the chocolate intakes performed.

Participants were randomised into two groups: intervention group (IG) and control group (CG). The randomisation sequence was carried out by an independent researcher using the Epidat 4.2 software [25]. Depending on the order of the baseline visit, participants received their randomisation number, which was hidden until all the women were assigned to a group. To ensure the blinding, the participants were clearly requested not to reveal their group to the blinded researchers during the interviews.

The characteristics of the intervention impeded the blinding of the participants. With the aim of minimising contamination between groups, the evaluation and chocolate resupply visits in the IG were conducted by different researchers. The information related to the randomisation of the treatment was kept in a safe in case of emergency unblinding.

### 2.5. Intervention

Participants in the CG did not receive any intervention, while participants in the IG were supplied with cocoa-rich chocolate (99%) and were instructed to consume 10 g daily of this supplement added to their habitual food intake. The intervention period was 6 months. During the first supply visit, the IG participants were provided with instructions about the consumption and storage of the chocolate supplement, recommending them to take the daily dose at the same time of the day. Additionally, women in the IG were asked to record the time of each daily intake in a calendar provided by the researchers, which was returned to them at each resupply visit.

The daily nutritional contribution of 10 g of this chocolate is 59 kcal, 0.8 g of carbohydrates, 1.5 g of protein and 5.1 g of fat, of which 3.1 g correspond to saturated fat. The contribution of polyphenols per 10 g of this product is 65.4 mg. All participants were asked to continue with their usual dietary pattern without modifying their eating habits during the study period.

### 2.6. Main Outcomes

Quality of life (QoL) was evaluated using two tools: the 3-level version of EuroQol-5D (EuroQoL-5D-3L) and the Cervantes scale.

EuroQol [26,27] is a health state measurement that includes two tools: the EuroQol-5D (EQ-5D) and the EuroQol Visual Analogue Scale (EQ-VAS). In the present study, the 3-level version of EQ-5D (EQ-5D-3L) was used, which descriptively analyses five dimensions (mobility, self-care, usual activities, pain and discomfort and anxiety and depression) on a scale of three possible answers categorised from 1 to 3 (1 “I have no problems”, 2 “I have some problems”, 3 “I have serious problems”). The results are presented through a descriptive analysis of each of the five dimensions and through the estimation of a general health state index between 0 (worst health state) and 1 (best health state) [28]. This EQ-5D summary index is derived by applying a formula that essentially attaches values (weights) to each of the levels in each dimension. The index can be calculated by deducting the appropriate weight from 1, the value for full health.

The EQ-VAS is a visual analogue scale that evaluates the general health state perceived by each individual. The range of this scale is between 0 (very bad health state) and 100 (optimal health state).

The Cervantes scale [29] was specifically designed for postmenopausal women. It assesses the impact of menopause on different physical and psychosocial characteristics and, especially, its effect on general wellbeing. It consists of 31 items structured in 4 dimensions: menopause and health, sexuality, mental domain and relationship. Furthermore, the dimension “menopause and health” includes three subdimensions: vasomotor symptoms, health and ageing. Regarding the global score of the scale, the highest score is 155 points, which represents low QoL, whereas the lowest score is 0 points, which represents the best QoL possible. All dimensions and subdimensions have a similar score range, where the lowest value indicates the best QoL and the highest value indicates the worst QoL.

### 2.7. Other Measurements

During the baseline evaluation, information about the clinical and sociodemographic variables was gathered. This information included the marital status, the education level and the time elapsed from the onset of menopause. Moreover, the history of the following morbidities was also recorded: gestational diabetes, hypertension and dyslipidaemia without pharmacological treatment and diagnosed depression. A more detailed description of the methodology and of the assessment of other variables, such as physical activity, alcohol consumption and smoking, is included in the trial protocol [21].

### 2.8. Data Collection Procedure, Data Management and Monitoring

The data collection in the evaluation visits was conducted by a previously trained nurse: this person was not the researcher in charge of making the randomisation and later analysis of the data. A unique identification code was used for every participant of the study in order to identify the data recorded in each measurement. Thus, a database was created, which could only be accessed by the professionals who worked in the study.

### 2.9. Ethical Considerations

The study was approved by the Clinical Research Ethics Committee of the Salamanca Health Area (‘CREC of the Health Area of Salamanca’) in February 2018 (ethic approval code: PI11812/2017). Informed consent was provided by each participant in accordance with the Declaration of Helsinki. All participants received information on the objectives of the project and the risks and benefits of the explorations to be conducted. The confidentiality of the participants’ data was guaranteed at all times in accordance with the provisions of Organic Law 3/2018, of 5 December, on Personal Data Protection and digital rights, and European Regulation 2016/679 of the European Parliament and of the Council of 27 April 2016 on General Data Protection (GDPR), and under the conditions established by Spanish Law 14/2007 on biomedical research.

### 2.10. Statistical Analyses

The statistical analyses were performed according to the study protocol [21]. The data were checked for normal distribution and most of the data were considered normally distributed. The characteristics of the study population are presented as mean and standard deviation (SD) or median (interquartile range) for the continuous variables and as frequency distribution for the qualitative variables. To evaluate the comparability in the baseline evaluation between the two groups, the chi-square test and Fisher’s exact test were used for the qualitative variables. In addition, a Student’s *t*-test and Mann–Whitney U test were applied to compare the mean values between groups. The effect of chocolate consumption on the main variables of QoL was analysed through an analysis of covariance (ANCOVA), adjusted for the main determinants of QoL gathered in the interviews: age, education level, marital status, time elapsed since the beginning of menopause, daily calorie intake (kcal), baseline consumption of chocolate with 70% cocoa, physical activity, alcohol consumption, smoking, depression and untreated dyslipidaemia. The results of these analyses are presented as estimated marginal mean and its 95% confidence interval (CI). The change in each group was analysed using a Student’s *t*-test for paired data. To address the possible bias due to a lack of response in some items of the Cervantes scale, we applied the multiple imputation by chained equations with 50 sets of data imputed to the results and covariables [30,31]. The estimations of each set of imputed data were combined following the guidelines described by Rubin [32]. An alpha risk of 0.05 was established as the limit of statistical significance. All analyses were performed using SPSS V.23.0 (IBM Corp, Armonk, NY, USA).

## 3. Results

### 3.1. Baseline Characteristics of the Study Participants

A total of 140 women (IG: *n* = 73, CG: *n* = 67) were included in this study. Three participants were lost in the evaluation at 6 months from the beginning of the study (IG: *n* = 2, CG: *n* = 1). The two losses in IG corresponded to two women who were diagnosed with cancer under pharmacological treatment during the study period, whereas the one from CG was a woman who decided to abandon the study in the follow-up visit (Figure 1). At baseline, no differences were found in any of the clinical or sociodemographic variables analysed (age, marital status or education level), nor in the time elapsed from the beginning of menopause. The baseline consumption of chocolate (g/week) was similar in both groups, as well as the rest of the analysed lifestyles: smoking, alcohol consumption, daily calorie intake and physical activity. Similarly, no differences were found in the baseline evaluation in terms of the number and percentage of women with a history of gestational diabetes, depression, untreated dyslipidaemia or hypertension (Table 1). Daily energy intake and nutrients of habitual diet, as well as chocolate intake, remained unchanged during the intervention in both groups.

### 3.2. Changes in QoL Measured with EuroQol

A descriptive analysis of the five dimensions evaluated in the EQ-5D-3L (mobility, self-care, usual activities, anxiety/depression and pain/discomfort) both in the baseline evaluation and at the 6-month evaluation visit is shown in Table 2. No differences were observed in the baseline evaluation between the groups in any of the dimensions.

In a model adjusted for the main determinants of QoL, the group that consumed 10 g of cocoa-rich chocolate daily showed a non-significant increase of 0.044 in the EQ-5D-3L score (95% CI: −0.012, 0.099) (*p* = 0.125), with respect to the CG. Moreover, the results of the EQ-VAS showed an increase of 6.0 (95% CI: 0.4, 11.7) in favour of the intervention group (*p* = 0.036) (Table 3).

### 3.3. Changes in QoL Measured with the Cervantes Scale

Using the same ANCOVA model adjusted for the main determinants of QoL considered in this study, no significant changes were observed in the global score of QoL evaluated with the Cervantes scale (1.1 points; 95% CI: −4.5, 6.7; *p* = 0.478). Similarly, no changes were found when analysing menopause and health, mental domain, sexual relations and relationship; likewise, the subdimensions of vasomotor symptoms, health and ageing did not show any significant differences (Table 4).

## 4. Discussion

The results of this clinical trial show that, in a sample of postmenopausal women, the daily consumption of 10 g of cocoa-rich chocolate produced a slight improvement in the score of the visual analogue scale of the EuroQol questionnaire (EQ-VAS), although there were no changes in the global score of QoL evaluated with the general questionnaire (EuroQoL-5D) or with a specific questionnaire for this population (Cervantes scale). Similarly, there were no changes in the dimensions or subdimensions analysed in the latter: menopause and health, mental domain, sexual relations, relationship, vasomotor symptoms, health and ageing.

The main tool used for the evaluation of health-related QoL in this study was the EuroQoL questionnaire. Within this questionnaire, there are two clearly differentiated sections with different purposes and objectives. The profile of the EQ-5D was developed to describe, in a quick and simple manner, the dimensions of health-related QoL (mobility, self-care, usual activities, pain and discomfort, anxiety and depression), as well as to estimate a single value that summarises all of these dimensions [26]. On the other hand, the EQ-VAS is aimed at obtaining the general health state of the participant, providing important and complementary information about the opinions of the patients regarding their own health. In a trial conducted in older individuals, an improvement in QoL measured with the EQ-5D was observed in the group that received a natural beverage made of flavonoid-rich cocoa powder, with a significant decrease in their perception toward problems in mobility and pain/discomfort, and without changes in the rest of the dimensions [33]. In our study, the proportion of women in each of the categories of the dimensions analysed in the EQ-5D-3L remained similar after the intervention. However, there were differences in the values of the EQ-VAS. The intake of chocolate is anecdotally associated with an increase of happiness, although few experimental studies have analysed this effect. Its sensory characteristics, nutritional composition and the presence of psychoactive components, such as tyramine and theobromine, have been suggested as the agents that cause the effects of chocolate on mental health and mood [34]. Another study revealed that chocolate seems to increase positive mood, particularly when eaten mindfully [35]. The EQ-VAS comprises all the aspects of health-related QoL and not only the content of the five dimensions measured in the EQ-5D. Therefore, the differences observed in the results of the EQ-VAS could help to value the general health status in further agreement with the perspective of the patient [36]. The results of the effect of a controlled and moderate dose of chocolate (10 g daily) in postmenopausal women observed in our study could be consistent with the effect attributed to chocolate on mood. The amount of 10 g of chocolate used in the intervention taken every day complies with the recommendations of the European Food Safety Authority [37] to maintain endothelium-dependent vasodilation [37], which is relevant since the main outcome of the trial that this study is part of was blood pressure, and Nurk et al. [38], who highlighted the attainment of a maximal beneficial effect on mental health with this daily amount of such product. Furthermore, it was reported that consumption of 14 g of milk chocolate enhanced positive mood [35]. Though the product used in this trial was dark chocolate, which is known to contain higher quantity of cocoa than milk chocolate, and the consumption of a similar amount of it was expected to have a greater effect on quality of life, it may not be sufficient to show changes in this outcome.

No changes were detected in the different dimensions of QoL evaluated with the Cervantes scale. This scale, validated in a Spanish population of perimenopausal women of 45–64 years of age [29], has been used in some studies [39,40]. The consumption of chocolate (including dark chocolate, milk chocolate and white chocolate) as a baseline condition in the study groups was 68 g/week (median (interquartile range): 42 (9–109) g/week in the IG and 50 (21–80) g/week in the CG), and this consumption was not restricted during the study. Therefore, the aim was to analyse whether an additional contribution of 10 g/day of dark chocolate improved the study variables. This was a condition of the design that could partly justify the absence of additional beneficial results on the dimensions of the Cervantes scale, especially on sexuality. In previous studies, the consumption of chocolate had a significant impact on sexual desire in women [11], although after adjusting for age, such association disappeared. However, it is necessary to further investigate the impact of the intake of chocolate on specific aspects of QoL.

Although it was slight, the change found in the score of the EQ-VAS in favour of the experimental group, which received the additional amount of dark chocolate, could reflect a better perception toward the general health state after the intervention. Considering the clinical context posed by a decrease of QoL after menopause, those interventions, especially the non-pharmacological ones, which can improve the health state could be clinically relevant. This is especially important in people over 60 years of age, in whom the changes in QoL are considered as potential predictors of mortality [41].

Chocolate is a natural source of caffeine. The amount of this component present in chocolate varies depending on the percentage of cocoa it contains [42]. Caffeine has multiple effects on health, some of which could affect quality of life. Among the effects exerted by caffeine, it has to be highlighted its potential vasoconstricting and anti-inflammatory effects, which can have a positive influence on pain relief [42]. Moreover, the benefits of chocolate on mood seem to be mainly exerted by caffeine [43]. Also, caffeine consumption could reduce sleep quality [44], which has been shown to be associated with hot flashes, loss of sexual interest and depressed mood [45], having a negative impact on quality of life. Nonetheless, our results did not show any significant change in these domains, and none of the questionnaires used explore sleep quality as a component of quality of life.

Modifications in the dietary pattern and eating habits usually followed by participants could have possibly altered the results, hence all subjects were asked to not modify these during the study period. Results showed that daily energy intake, nutrients of habitual diet and chocolate intake remained unchanged in both the IG and CG, which suggests that these parameters were not altered during the intervention.

Another point to discuss is the mean time from menopause onset, which was 6.9 years in both study groups. This has to be considered, as many of the physiological changes during menopause take place during the period of menopausal transition. Avis et al. observed that the duration of vasomotor symptoms was more than 7 years during the menopausal transition for more than half of the participants in the Study of Women’s Health Across the Nation (SWAN), and persisted for 4.5 years after the final menstrual period [46]. These vasomotor symptoms are associated with poorer quality of life, negative mood and sleep problems [47]. Considering this, carrying out this trial earlier in the menopause period could have had a greater impact on quality on life and should be taken into account in future research.

There are some limitations that must be highlighted. Firstly, the design of the trial that this study is part of does not establish the improvement of QoL as the main objective, and it is taken as a secondary variable. Thus, the sample size could be insufficient for the contrast of this variable. Secondly, there was no blinding in the participants due to the nature of the intervention, which may have influenced the findings about QoL. The participants of the study had no restrictions on the consumption of cocoa or chocolate in terms of presentation, which may underestimate the effect of the intervention; however, this approach is more in line with the eating habits of the population. Another limitation could be the lack of control of polyphenol intake during the intervention. This was not possible since the tool used to assess the nutritional composition of the habitual diet does not provide data on the polyphenol content of the diet or the specific foods consumed. We could assume that randomisation had balanced the groups with respect to dietary intake as well as polyphenol intake; nonetheless, this should be considered in future studies.

## 5. Conclusions

We can conclude that the additional daily contribution of 10 g of cocoa-rich chocolate in postmenopausal women could have a slight impact on their perception toward their health state, although without modifying the health-related QoL or the dimensions that compose it. Despite this, further analytical studies are required to delve specifically into these associations.

## Figures and Tables

**Table 1:** Baseline characteristics of postmenopausal women participants in the study.

	Intervention Group (*n* = 73)	Control Group (*n* = 67)
Age, years	57.1 ± 3.5	57.5 ± 3.8
Marital status, *n* (%)		
Married/cohabitant	48 (65.8)	47 (70.1)
Separated/divorced	8 (11.0)	7 (10.4)
Single	15 (20.5)	9 (13.4)
Widowed	2 (2.7)	4 (6.0)
Education level, *n* (%)		
Elementary education	16 (21.9)	12 (17.9)
Middle–High school	22 (30.1)	29 (43.3)
Bachelor	17 (23.3)	11 (16.4)
Postgraduate	18 (24.7)	15 (22.4)
Time from menopause onset, years	6.9 ± 4.6	6.9 ± 3.6
Chocolate intake, g/week	42 (9–109)	50 (21–80)
>70% cocoa chocolate intake, g/week	0 (0–26)	0 (0–24)
Lifestyles		
Smokers, *n* (%)	12 (16.4)	9 (13.4)
Adequate alcohol consumption, *n* (%)	71 (97.3)	65 (97.0)
Physical activity light intensity, MET h/week	18.5 ± 9.4	18.8 ± 11.3
Energy, kcal/day	1720 ± 357	1780 ± 402
Morbidities, *n* (%)		
Untreated dyslipidaemia	8 (11.0)	10 (14.9)
Untreated hypertension	1 (1.4)	0 (0)
Gestational diabetes	3 (4.1)	1 (1.5)
Depression	15 (20.5)	15 (22.4)

Values expressed as mean ± standard deviation, median (interquartile range) or frequencies. Abbreviations: MET, metabolic equivalent of task.

**Table 2:** EQ-5D-3L variables in postmenopausal women participants.

	Intervention Group (*n* = 71)		Control Group (*n* = 66)	
	Baseline	6 Months	Baseline	6 Months
Mobility, *n* (%)				
No problems in walking about	65 (91.5)	69 (97.2)	62 (93.9)	60 (90.9)
Some problems in walking about	6 (8.5)	2 (2.8)	4 (6.1)	6 (9.1)
Confined to bed	-	-	-	-
Self-care, *n* (%)				
No problems with self-care	71 (100)	71 (100)	66 (100)	66 (100)
Some problems washing or dressing herself	-	-	-	-
Unable to wash or dress herself	-	-	-	-
Usual activities, *n* (%)				
No problems with performing her usual activities	66 (93.0)	69 (97.2)	65 (98.5)	63 (95.5)
Some problems with performing her usual activities	5 (7.0)	2 (2.8)	1 (1.5)	3 (4.5)
Unable to perform her usual activities	-	-	-	-
Anxiety and depression, *n* (%)				
Not anxious or depressed	51 (71.8)	55 (77.4)	57 (86.4)	59 (89.4)
Moderately anxious or depressed	19 (26.8)	15 (21.1)	9 (13.6)	7 (10.6)
Extremely anxious or depressed	1 (1.4)	1 (1.4)	-	-
Pain and discomfort, *n* (%)				
No pain or discomfort	46 (64.8)	52 (73.2)	50 (75.8)	46 (69.7)
Moderate pain or discomfort	23 (32.4)	19 (26.8)	15 (22.7)	20 (30.3)
Extreme pain or discomfort	2 (2.8)	-	1 (1.5)	-

Abbreviations: EQ-5D-3L, 3-level version of EQ-5D.

**Table 3:** Changes in the EQ-5D-3L score and EQ-VAS in postmenopausal women participants.

	Intervention Group (*n* = 71)		Control Group (*n* = 66)			
	Baseline	6 Months	Baseline	6 Months	Adjusted Intergroup Difference (IG-CG) ^1^	***p***
EQ-5D-3L ^2,3^	0.868 ± 0.159	0.901 ± 0.123	0.919 ± 0.124	0.907 ± 0.124	0.044 (−0.012, 0.099)	0.125
EQ-VAS ^4^	74.7 ± 13.9	78.1 ± 14.0	78.3 ± 13.6	77.2 ± 14.9	6.0 (0.4, 11.7)	0.036

Values expressed as mean ± standard deviation. Abbreviations: EQ-5D-3L, 3-level version of EQ-5D; EQ-VAS, EuroQoL visual analogue scale. ^1^ These values are adjusted for age, education level, marital status, time elapsed since the beginning of menopause, daily calorie intake (kcal), baseline consumption of chocolate with 70% cocoa, physical activity, alcohol consumption, smoking, depression and untreated dyslipidaemia. Results are based on analysis of covariance (ANCOVA). ^2^ Difference between groups at baseline (*p* < 0.05). ^3^ Range between 0 (worst quality of life) and 1 (best quality of life). ^4^ Range between 0 (worst quality of life) and 100 (best quality of life).

**Table 4:** Changes in the Cervantes scale score and dimensions in postmenopausal women participants.

	Intervention Group (IG) (*n* = 71)		Control Group (CG) (*n* = 66)			
	Baseline	6 Months	Baseline	6 Months	Adjusted Intergroup Difference (IG–CG) ^1^	*p*
Total score (0–155) ^2^	51.8 (2.5)	52.0 (2.4)	48.5 (2.4)	47.4 (2.5)	1.1 (−4.5, 6.7)	0.478
**Dimensions**						
**Menopause and health (0–75)**	26.2 (1.5)	26.1 (1.4)	24.9 (1.4)	24.9 (1.3)	0.1 (−2.8, 3.2)	0.927
Vasomotor symptoms (0–15)	6.8 (0.6)	6.5 (0.5)	6.3 (0.6)	5.7 (0.5)	0.3 (−0.8, 1.4)	0.622
Health (0–25)	7.7 (0.6)	8.0 (0.6)	7.8 (0.5)	7.8 (0.5)	0.3 (−1.0, 1.6)	0.652
Ageing (0–35)	11.7 (0.7)	11.6 (0.6)	10.8 (0.7)	11.3 (0.7)	−0.6 (−2.1, 0.9)	0.431
**Mental domain (0–45)**	8.5 (0.9)	9.6 (0.9)	8.2 (0.7)	8.3 (0.9)	1.0 (−1.1, 3.0)	0.357
**Sexuality (0–20)**	11.4 (0.5)	10.7 (0.6)	11.2 (0.6)	10.2 (0.6)	0.2 (−1.5, 1.8)	0.848
**Relationship (0–15)**	5.7 (0.6)	5.7 (0.6)	4.7 (0.6)	4.3 (0.6)	−0.2 (−1.5, 1.1)	0.775

Values expressed as mean (standard error) and differences are means (95% confidence interval). ^1^ These values are adjusted for age, education level, marital status, time elapsed since the beginning of menopause, daily calorie intake (kcal), baseline consumption of chocolate with 70% cocoa, physical activity, alcohol consumption, smoking, depression and untreated dyslipidaemia. Results are based on analysis of covariance (ANCOVA). ^2^ Range between 0 (best quality of life) and 155 (worst quality of life).
